# Impact of Deuteration and Temperature on Furan Ring Dynamics

**DOI:** 10.3390/molecules26102889

**Published:** 2021-05-13

**Authors:** Przemyslaw Dopieralski, Iryna V. Omelchenko, Zdzislaw Latajka

**Affiliations:** 1 Faculty of Chemistry, University of Wroclaw, Joliot–Curie 14, 50-383 Wroclaw, Poland; zdzislaw.latajka@chem.uni.wroc.pl; 2 STC, Institute for Single Crystals, National Academy of Sciences of Ukraine, 60 Nauky Ave., 61001 Kharkiv, Ukraine; 3 Faculty of Chemistry, V.N. Karazin Kharkiv National University, 4 Svobody Sq., 61077 Kharkiv, Ukraine

**Keywords:** isotope effect, computational chemistry, aromaticity, furan, puckering

## Abstract

Despite significant progress in conformational analysis of cyclic molecules, the number of computational studies is still limited while most of that available in the literature data have been obtained long time ago with outdated methods. In present research, we have studied temperature driven conformational changes of the furan ring at three different temperatures. Additionally, the effect of deuteration on the ring dynamics is discussed; in addition, the aromaticity indices following the Bird and HOMA schemes are computed along all trajectories. Our ab initio molecular dynamic simulations revealed that deuteration has changed the furan ring dynamics and the obvious consequences; in addition, the shape and size of molecule are expected to be different.

## 1. Introduction

Isotope effect and aromaticity are the key concepts in modern organic chemistry [1,2,3,4,5] with H/D isotope substitution being one of the fundamental aspects of NMR labeling, Neutron Scattering, and Mass Spectroscopy [6,7,8,9,10].

Furan is a cyclic aromatic ether found in cigarette smoke and used in the production of resins [11] and some agrochemicals and pharmaceuticals. Obtaining furan from “green” inedible lignocellulosic biomass is one of the promising sustainable, and industrially applicable alternatives to various petroleum-derived chemicals [12,13]. Recently, investigations on the presence of furan in coffee, juices, soups, and canned fruits or vegetables has gained widespread attention [14,15,16].

It is said that the presence of an aromatic conjugated system is responsible for bond equalization and most importantly the planarity of the ring. These statements seem to be in agreement with many experimental and theoretical studies [17,18,19] Therefore, for many decades, rings with aromatic character have been considered as flat and conformationally rigid molecules, until the recent theoretical studies [20,21,22,23] based on ab initio calculations, both at T=0K and with T>0K, that have shown the large amount of flexibility of aromatic molecules.

Our previous studies on isolated benzene molecule at 298 K exposed the fact that the population of this aromatic ring with planar geometry is very low (less than 7%). The molecule exists basically as a set of two families of flattened boat and half-boat conformations, which have average values of endocyclic dihedral angles of 7${}^{\circ }$, which allowed for explaining the results of experimental electronic spectra. Due to the high amount of symmetry of benzene molecules, electronic transitions should be forbidden However, out-of-plane deformations of benzene rings decrease the symmetry, allowing electronic transitions [24,25,26]. In light of these results, we asked the following questions: what is happening with the planarity of aromatic heterocyclic five–membered rings (i.e., furan), how does the temperature affect its dynamics, and how does deuteration of the ring influence its planarity?

Finally, we would like to point out that two competing mechanisms of odor detection—chemical and the spectral—are usually investigated by deuteration of odorants; see [27,28,29,30,31,32,33] for reference. However, our research exposed different perspectives on the influence of deuteration on the ring dynamics and thus also “volume/shape” of the molecule studied [34].

## 2. Results and Discussion

The full conformational space of five–membered ring includes pure 10 Envelope (*E*) and 10 Twist (*T*) conformations (and their intermediates) [35,36,37,38]. However, in our case, they can be reduced to six unique conformations due to the symmetry of the furan ring—see Supplementary Materials for the conformational circle.

Each single trajectory spanned around 100 ps, and, to asses changes in aromaticity, we have calculated two commonly used indices following the Bird and HOMA schemes [19,39,40] for each single step over all the trajectories. It is shown in Table 1.

It is seen that aromaticity decreases with growing temperature in both isotopomers, and the values of Bird and HOMA parameters are quite similar in two molecules at the same temperature. There is only one notable difference in the distribution (range) of values in both indices, it is wider for deuterofuran (FuD) at 50 K, and 300 K, but narrower at 500 K. It enables us to hypothesize that deuteration could be responsible for hampering the vibrations with maximal shortening or elongation of bonds.

**Figure 1:**
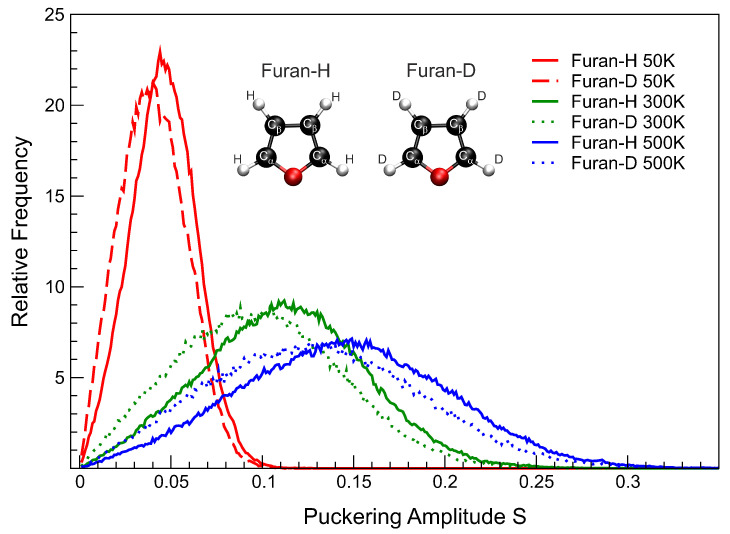
Relative frequency of puckering amplitude for furan (solid lines, furan-H) compared with deuterofuran (broken lines, furan-D) at three different temperatures: 50 K, 300 K, and 500 K derived from CPMD simulations. Inset represents studied molecules together with atoms labeling.

Ring puckering parameters *S* defining degree of non–planarity and phase angle defining conformation type [41] were calculated for each single step of all trajectories. The frequencies of puckering amplitudes of the furan ring *S* obtained from our simulations at 50 K, 300 K and 500 K are shown in Figure 1. It is found that mean values of puckering amplitude *S* in deuterofuran are systematically lower as compared to furan in all cases. In addition, we observed a slight asymmetry in distribution over *S* for deuterofuran. At all temperatures, the median values are shifted to the area of smaller *S* as compared to furan, whilst the distribution width is almost the same. Thus, deuterofuran reveals long tails in the area of bigger *S* values. Therefore, the presence of D atoms makes the out-of-plane ring deformation less favorable, but the higher limit of flexibility is at the same level as in furan.

Simulations at the room temperature revealed that the population of planar conformation of the furan ring is around 39.5%, which is much higher than that for benzene (cf. 6.6%), pyrimidine, or 1,2,4–triazine—cf. 30.1% and 26.7%, respectively [22]. However, this is to be expected since smaller cyclic molecules are geometrically more rigid. On the other hand, furan has much less aromatic character than benzene, and the population of planar geometry is much larger, which compels one to ask whether the concepts of aromatic organic chemistry still make sense in the light of the presented results.

**Figure 2:**
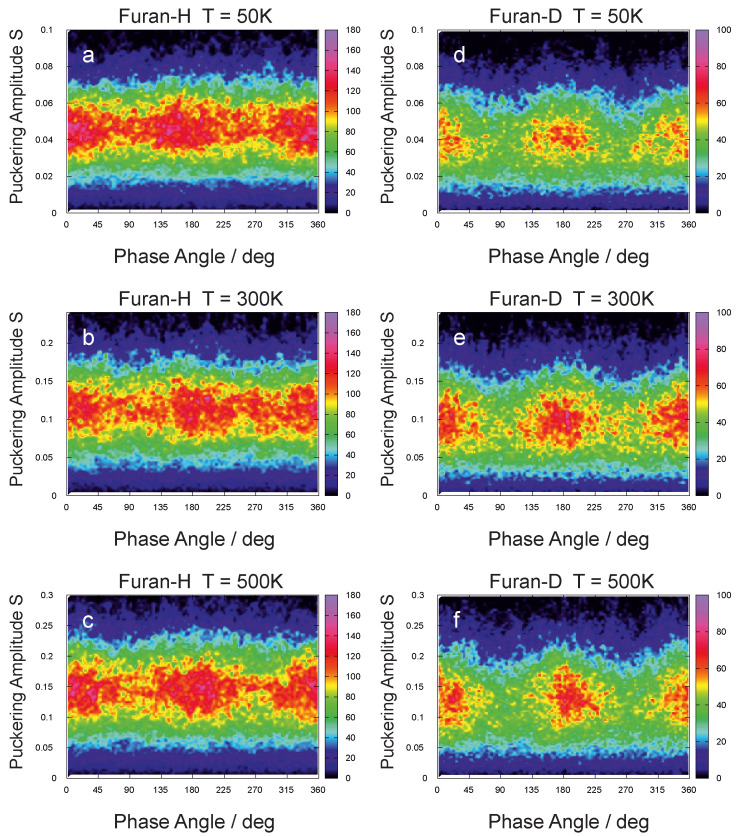
Gradient maps over the puckering amplitude *S* and phase angle ψ, at three different temperatures: 50 K, 300 K, and 500 K derived from CPMD simulations for furan (furan–H) and deuterofuran (furan–D).

Further analysis has found the distribution over the phase angle to be homogeneous in furan, and, surprisingly, it corresponds for deuterofuran to be more localized at three phase angles—0${}^{\circ }$, 180${}^{\circ }$ and 360${}^{\circ }$, see Figure 2.

Thus, only deuterofuran tends to keep preferable conformations, even at higher temperatures, while furan does not demonstrate such localization and moves freely over all conformational space. The concurrent analysis of both puckering parameters did not reveal any significant difference between envelope and twist conformations in the areas of preferable conformations. All conformations that lead the oxygen atom out of the ring plane (first of all, Envelope O and Twist O–C${}_{\alpha }$) are more favorable than conformations that leave oxygen atoms in the plane.

**Figure 3:**
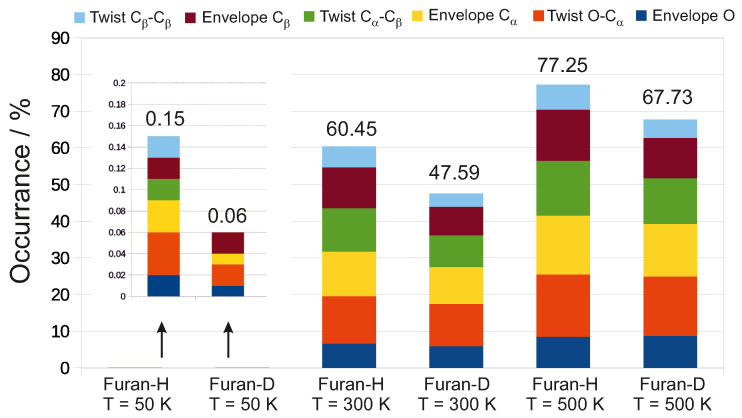
Furan and deuterofuran population of non-planar geometries decomposed into six different conformations: twist C${}_{\beta }$–β (blue), envelope C${}_{\beta }$ (brown), twist C${}_{\alpha }$–β (green), envelope C${}_{\alpha }$, twist O–C${}_{\alpha }$ (red) and envelope O (dark blue) at three different temperatures: 50 K, 300 K, and 500 K derived from CPMD simulations. Non-planar populations at 50 K have been enlarged for visibility.

The results of simulations show that the population of planar geometry for furan (deuterofuran) decreases significantly with the increase of the temperature: 99.85 (99.94)% versus 22.75 (32.27)% from 50 K to 500 K. The populations of six non–planar conformations are shown in Figure 3.

## 3. Materials and Methods

In order to investigate the conformational dynamics of furan and deuterated furan, we performed molecular dynamic calculations using ab initio molecular dynamics [42] using the efficient Car–Parrinello [43] propagation scheme as implemented in the CPMD program package [44]. The simulations were performed in the canonical ensemble at 50, 298, and 500 K. To control the temperature of the system, the NHC thermostat [45] was turned on and set at a frequency of 3000 (2500) cm${}^{-1}$ for furan (deuterated furan). A molecular dynamics time step of Δt=2 a.u. (Δt=4 a.u.)—≈ 0.097 (≈ 0.048) fs—was used for the integration of the Car–Parrinello equations of motion using a fictitious mass parameter for the orbitals of 400 (700) a.u. together with the proper atomic masses for furan (deuterated furan). The supercell was a cubic box of 20.0 Å in length, and cluster boundary conditions were applied to properly treat the isolated system.

Conformational analysis was performed following the scheme developed by Zefirov, Palyulin, and Dashevskaya [41] that uses torsion angles instead of RMS displacements of atoms within the classical Cremer–Pople puckering formalism [35], in order to bypass some shortcomings of Cremer–Pople method related to non-equal bond lengths. There are a couple of different schemes for calculating puckering parameters; however, the difference between these methods is negligible for common heterocyclic rings like THF [46,47] and is likely negligible for furan. There are only two puckering parameters for a five-membered ring, one phase angle ψ that defines conformation, and puckering amplitude *S* that defines the degree of planarity of the ring. The phase angle ϕ that defines conformation and the puckering amplitude *S* that defines the degree of planarity of the ring are presented in the Supplementary Materials. For more details about the procedure used, please see [35,41] or the most recent paper by Chan [48].

For more details and equations, please see Supplementary Materials.

## 4. Conclusions

In this study, we investigated furan ring dynamics at three different temperatures also taking into account the effect of deuteration on ring puckering. It was found that furan is much more rigid than benzene and aromatic azines, while its mean degree of aromaticity is much lower. In addition, there is no strict relation: aromaticity vs. conformational dynamics of furan and deuterofuran at different temperatures. While degree of aromaticity is similar for both molecules, there are obvious differences in conformational behavior. It seems that, for molecules possessing an aromatic π-system, the particular degree of aromaticity does not simply predict the conformational dynamics and degree of non-planarity, especially at higher temperatures—other structural and dynamic conditions affect the activation of out-of-plane modes much more than aromatic π-conjugation. It is shown that a deuterated furan ring behaves differently in comparison to furan with puckering amplitude *S* being smaller, which strongly suggests that non-deuterated carbon atoms are ”volumetrically” larger. This is a very important conclusion that contradicts standard experimental assumptions that deuteration is leaving the size of molecules unchanged.

## Figures and Tables

**Table 1:** Aromaticity indices for furan (FuH) and deuterofuran (FuD) at three temperatures: 50 K, 300 K, and 500 K.

	I5			HOMA		
	Range	Mean	Median	Range	Mean	Median
FuH 50 K	38.3	46.7	46.7	1.09	0.14	0.15
FuH 300 K	104.7	41.9	41.9	3.42	−0.10	−0.06
FuH 500 K	122.2	37.8	38.0	4.44	−0.32	−0.24
FuD 50 K	42.6	46.7	46.7	1.15	0.14	0.15
FuD 300 K	105.3	42.0	42.0	3.68	−0.10	−0.06
FuD 500 K	113.6	37.9	38.0	4.36	−0.32	−0.25

## Data Availability

Not applicable.

## Supplementary Materials

The following are available online. Figure S1: The full conformational space of furan ring: pure 10 Envelopes (E) and 10 Twist (T) conformations and their intermediates. It is reduced to only 6 unique conformations due to the symmetry of furan ring see populations to all of them in Table S1, Figure S2: Distribution of I${}_{5}$ indexes for furan and deuterofuran at three temperatures 50 K, 300 K and 500 K. The distributions at the same temperature for H-Furan and D-Furan are very similar and they simply overlap and to distinguish between them the y-scale has been changed that going up and down from ’0’ always positive values are shown and D-Furan is shown at bottom panel, Figure S3: Distribution of HOMA indexes for furan and deuterofuran at three temperatures 50 K, 300 K and 500 K. See description of Figure S2, Table S1: Population in % of all six conformations of the furan ring obtained from *ab initio* molecular dynamics at three temperatures 50 K, 300 K and 500 K. Note that the Envelope O and Twist C${}_{\beta }$–-C${}_{\beta }$ conformations are doubly degenerated while all other are 4-fold degenerated, thus in order to compare the probabilities one must divide the percentage of each 4-fold degenerated conformations by 2.
