# Supplementary material for: Impact of Deuteration and Temperature on Furan Ring Dynamics
Source: Molecules. 2021 May 13;26(10):2889. doi: 10.3390/molecules26102889 (PMC8152745; doi:10.3390/molecules26102889)
Supplement: Supplementary file 1 [file molecules-26-02889-s001.zip › molecules-1192434-supplementary.pdf]

Supporting Information:

Impact of Deuteration and Temperature on Furan Ring Dynamics

Przemyslaw Dopieralski[(1)],[*] Iryna V. Omelchenko[(2)],[†] and Zdzisław Latajka[(1)]

[(1)] *Faculty of Chemistry,*

University of Wroclaw, Joliot–Curie 14,

50-383 Wroclaw, Poland

[(2)] *STC, Institute for Single Crystals,*

National Academy of Sciences of Ukraine,

60 Nauky Ave., Kharkiv 61001, Ukraine

V.N.Karazin Kharkiv National University,

4 Svobody sq., Kharkiv 61077, Ukraine

Contents

I. Computational Details 3

A. Ab Initio Molecular Dynamics Simulations 3

B. Conformational analysis 4

References 9

I. COMPUTATIONAL DETAILS

A. Ab Initio Molecular Dynamics Simulations

In order to investigate the conformational dynamics of furan and deuterated furan, we performed molecular dynamic calculations using *ab initio* molecular dynamics[1] using the efficient Car–Parrinello[2] propagation scheme as implemented in the `CPMD` program package.[3] Simulations were carried out using the PBE1PBE/aug–cc–pVTZ optimized geometry in *GAUSSIAN*[4], and reoptimized using plane–wave basis set with kinetic energy cutoff of 100 Ry as a starting point to molecular dynamic simulations.

Following the initial equilibration period – 2 ps – where the temperature was controlled by a simple algorithm based on initializing the kinetic energy and then rescaling the velocities of the atoms whenever the instantaneous temperature were more than 50K away from the target temperature. After next step where each degree of freedom had a separate Nosé–Hoover-chain (NHC) thermostat[5], the data were collected over trajectories spanning a. 100 *ps*. Calculations were carried out using plane-wave basis and Γ–point sampling of the Brillouin zone. The Troullier-Martins normconserving pseudopotentials[6], the Becke et al. (PBE) exchange and correlation functional within the spin–restricted Kohn–Sham formalism was used. The simulations were performed in the canonical ensemble at 50, 298 and 500 K. To control the temperature of the system, the NHC thermostat was turned on and set at a frequency of 3000 (2500) cm^{-1} for furan (deuterated furan). A molecular dynamics time step of $\Delta t = 2$ a.u. ($\Delta t = 4$ a.u.) – ≈ 0.097 (≈ 0.048) fs – was used for the integration of the Car-Parrinello equations of motion using a fictitious mass parameter for the orbitals of 400 (700) a.u. together with the proper atomic masses for furan (deuterated furan). The supercell was a cubic box of 20.0 $\mathring{A}$ in length, and cluster boundary conditions were applied to properly treat the isolated system.
The VMD[7] (Visualize Molecular Dynamics) program has been used for data visualization.

B. Conformational analysis

Conformational analysis was performed following the scheme developed by Zefirov, Palyulin and Dashevskaya[8] that uses torsion angles instead of RMS displacements of atoms within the classical Cremer–Pople puckering formalism[9], in order to bypass some shortcomings of Cremer–Pople method related to non-equal bond lengths. There are a couple of different schemes for calculating puckering parameters; however, difference between these methods is negligible for common heterocyclic rings like THF[10,11] and is likely negligible for furan. There are only two puckering parameters for a five–membered ring, one phase angle ψ that defines conformation, and puckering amplitude S that defines the degree of planarity of the ring. They were calculated for each configuration in the simulations and are reduced for the particular case of five-membered ring to the following form:

$$S\cos(\psi) = -\sqrt{\frac{2}{5}}\sum_{j=1}^{5}\sin(\frac{\phi_j}{2})\sin(\frac{2\pi(2j+1)}{5})$$

$$S\sin(\psi) = -\sqrt{\frac{2}{5}}\sum_{j=1}^{5}\sin(\frac{\phi_j}{2})\cos(\frac{2\pi(2j+1)}{5})$$

where ϕ are torsion angles and j are numbering the corresponding torsion angles in the molecule (*j=1 for 1-2-3-4 angle, j=2 for 2-3-4-5.* Than values of $\tan(\psi)$ were calculated using these equations,

$$\tan(\psi) = \frac{\sum_{j=1}^{5}\sin(\frac{\phi_j}{2})\cos(\frac{2\pi(2j+1)}{5})}{\sum_{j=1}^{5}\sin(\frac{\phi_j}{2})\sin(\frac{2\pi(2j+1)}{5})}$$

and S^{init} values were calculated as:

$$S^{init} = \frac{(-\sqrt{\frac{2}{5}}\sum_{j=1}^{5}\sin(\frac{\phi_j}{2})\sin(\frac{2\pi(2j+1)}{5})}{\cos(\arctan(\tan(\psi)))}, \quad if \quad \cos(\arctan(tan(\psi))) > 0.01$$

$$S^{init} = \frac{(-\sqrt{\frac{2}{5}}\sum_{j=1}^{5}\sin(\frac{\phi_j}{2})\cos(\frac{2\pi(2j+1)}{5})}{\sin(\arctan(\tan(\psi)))}, \quad if \quad \cos(\arctan(tan(\psi))) < 0.01$$

Since arctan and *sin*/*cos* functions have different periods, final S and ψ values were calculated from $\tan(\psi)$ and S^{init} values taking into account that S amplitude should always be nonnegative.

$$\psi = \arctan(\tan(\psi)), S = S^{init} if S^{init} > 0$$

$$\psi = \arctan(\tan(\psi + \pi)), S = -S^{init} if S^{init} \leqslant 0$$

Atom numbering $O_1 - C_2 - C_3 - C_4 - C_5$ was kept unchanged in all configurations in the simulation run. If value of S is less than 0.1 then values of all the endocyclic torsion angles do not exceed ±5°. Therefore, conformations with $S \leq 0.1$ may be considered as planar or almost planar. We used this limit of S similarly as in our previous study of six–membered rings[12–14], however, we are not directly comparing absolute values of flexibilities based on this estimation for five–membered and six–membered ring, since smaller rings are geometrically more rigid.

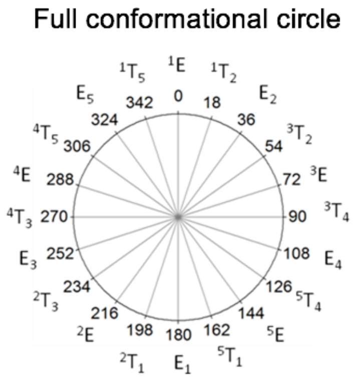

Conformation	Merged conformations	Merged phase angles	
Envelope O	^1E E_1	0 180	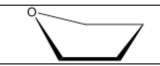
Twist O–Cα	$^1T_2$ $^5T_1$ $^2T_1$ $^1T_5$	18 162 198 342	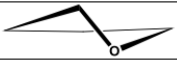
Envelope Cα	E_2 ^5E ^2E E_5	36 144 216 324	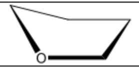
Twist Cα–Cβ	$^3T_2$ $^5T_4$ $^2T_3$ $^4T_5$	54 126 234 306	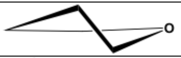
Envelope Cβ	^3E E_4 E_3 ^4E	72 108 252 288	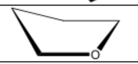
Twist Cβ–Cβ	$^3T_4$ $^4T_3$	90 270	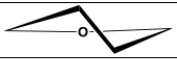

FIG. S1: The full conformational space of furan ring: pure 10 Envelopes (E) and 10 Twist (T) conformations and their intermediates. It is reduced to only 6 unique conformations due to the symmetry of furan ring - see populations to all of them in Table S1

Furan aromaticity is predictable low, I_5 index at temperature 50 K is very close to index I_5 calculated with "static" MP2/cc–pVTZ approach[15], thus 0 K and experimental data (51% and 49%, respectively)[16] for both molecules.

The I_5 distribution remains symmetrical (see Figure S2). Index HOMA at 0 K is considerably higher than predicted by our CPMD simulations (0.31 and 0.23 from MP2/cc–pVTZ and experiment, respectively). Actually the HOMA index seem to be systematically low for all O–containing heterocycles and most probably that could be a problem of HOMA

TABLE S1: Population in % of all six conformations of the furan ring obtained from *ab initio* molecular dynamics at three temperatures 50 K, 300 K and 500 K. Note that the Envelope O and Twist C_β—C_β conformations are doubly degenerated while all other are 4-fold degenerated, thus in order to compare the probabilities one must divide the percentage of each 4-fold degenerated conformations by 2

**Conformation**	*D 50*	*D 300*	*D 500*	*H 50*	*H 300*	*H 500*
Envelope O	0.01	5.96	8.75	0.02	6.61	8.48
Twist O—C_α	0.02	11.53	16.19	0.04	12.94	17.02
Envelope C_α	0.01	10.04	14.33	0.03	12.17	16.06
Twist C_α—C_β	0.00	8.63	12.50	0.02	11.81	14.99
Envelope C_β	0.02	7.83	10.99	0.02	11.17	13.88
Twist C_β—C_β	0.00	3.60	4.97	0.02	5.74	6.81
Total non-planar	0.06	47.59	67.73	0.15	60.45	77.25
Total planar	99.94	52.41	32.27	99.85	39.55	22.75

parameterization that assumes too small optimal value for aromatic bond length, which is 1.265 Å. Furan and deuterofuran reveal the very same behavior with the only notable difference is the range of values of indices, which is higher in D-derivative at 50K but lower at 500K.

Index HOMA drops with growing temperature much faster than I_5 and that is expected as HOMA considers also non-alternating bong lengths change. I_5 loses only 9% on the road from 50K to 500K but HOMA loses 46% with respect to fully aromatic 5-m ring. So the contribution of non-alternating bond lengths dominates in this case. Also, the mean and median values are significantly different at 300 K and 500 K in case of HOMA because of the asymmetry of the distribution(see Figure S3). The long tails going into negative values of HOMA were also observed in all previous simulations of benzene.

The asymmetry markedly increases from 50 K to 500 K together with decreasing of the mean value of HOMA.

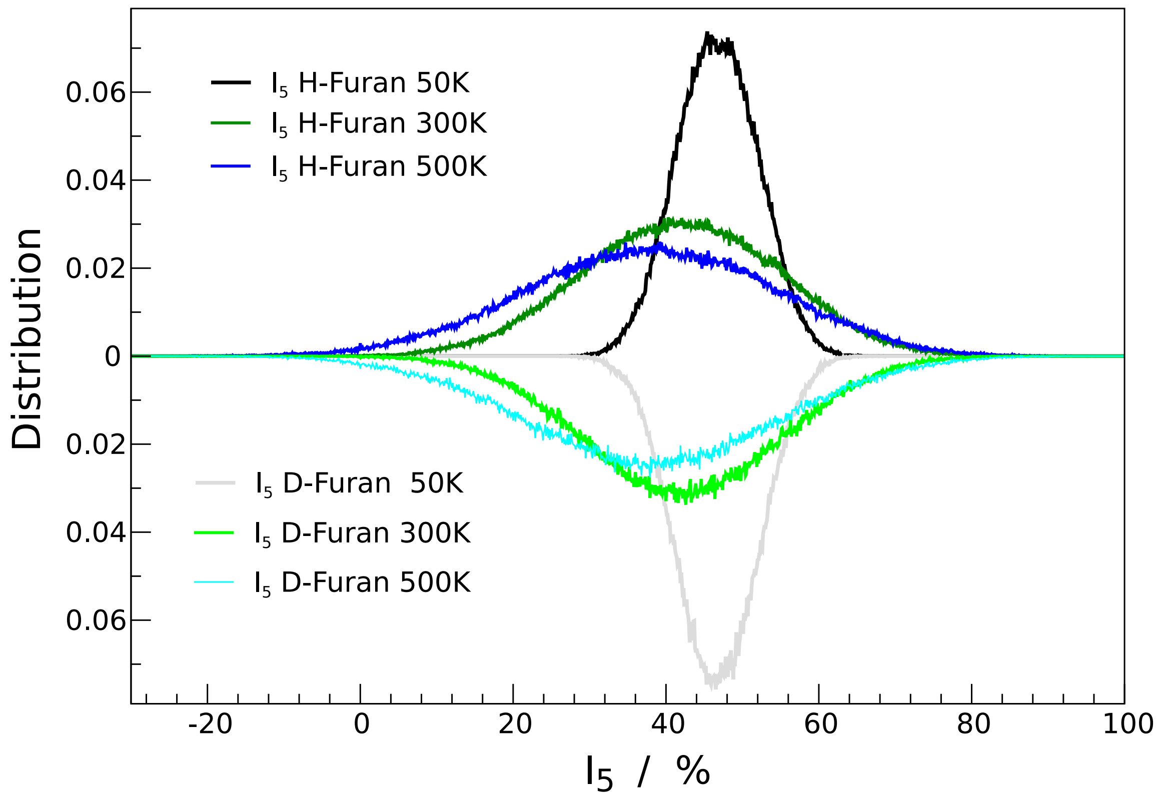

FIG. S2: Distribution of I_5 indexes for furan and deuterofuran at three temperatures 50 K, 300 K and 500 K. The distributions at the same temperature for H-Furan and D-Furan are very similar and they simply overlap and to distinguish between them the y-scale has been changed that going up and down from '0' always positive values are shown and D-Furan is shown at bottom panel.

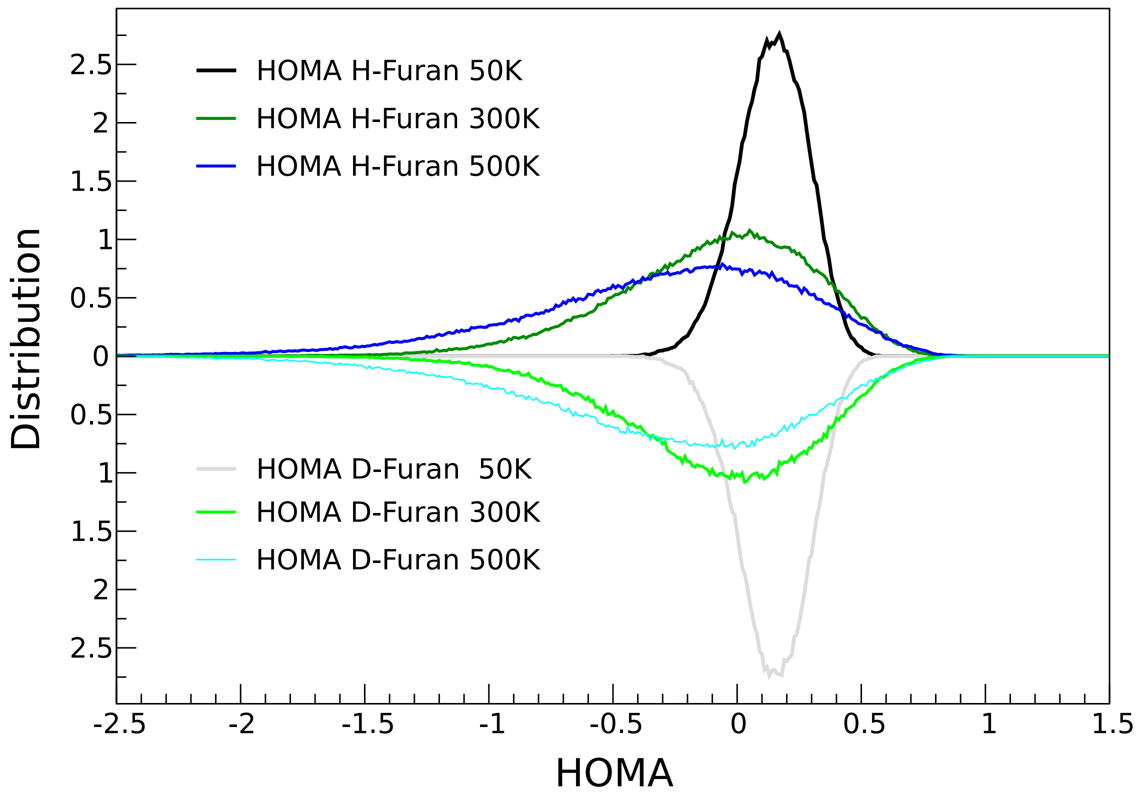

FIG. S3: Distribution of HOMA indexes for furan and deuterofuran at three temperatures 50 K, 300 K and 500 K. See description of Figure S2 in SI.

[*] Electronic address: przemyslaw.dopieralski@chem.uni.wroc.pl

[†] Electronic address: irina@xray.isc.kharkov.com

[1] D. Marx, J. Hutter, *Ab Initio Molecular Dynamics: Basic Theory and Advanced Methods*, Cambridge University Press, Cambridge, **2009**.

[2] R. Car, M. Parrinello, *Phys. Rev. Lett.* **1985**, *55*, 2471–2474.

[3] J. Hutter, *et al.*, CPMD *Program Package*, see http://www.cpmd.org.

[4] M. J. Frisch, G. W. Trucks, H. B. Schlegel, G. E. Scuseria, M. A. Robb, J. R. Cheeseman, G. Scalmani, V. Barone, B. Mennucci, G. A. Petersson, H. Nakatsuji, M. Caricato, X. Li, H. P. Hratchian, A. F. Izmaylov, J. Bloino, G. Zheng, J. L. Sonnenberg, M. Hada, M. Ehara, K. Toyota, R. Fukuda, J. Hasegawa, M. Ishida, T. Nakajima, Y. Honda, O. Kitao, H. Nakai, T. Vreven, J. A. Montgomery, J. E. Peralta, F. Ogliaro, M. Bearpark, J. J. Heyd, E. Brothers, K. N. Kudin, V. N. Staroverov, R. Kobayashi, J. Normand, K. Raghavachari, A. Rendell, J. C. Burant, S. S. Iyengar, J. Tomasi, M. Cossi, N. Rega, J. M. Millam, M. Klene, J. E. Knox, J. B. Cross, V. Bakken, C. Adamo, J. Jaramillo, R. Gomperts, R. E. Stratmann, O. Yazyev, A. J. Austin, R. Cammi, C. Pomelli, J. W. Ochterski, R. L. Martin, K. Morokuma, V. G. Zakrzewski, G. A. Voth, P. Salvador, J. J. Dannenberg, S. Dapprich, A. D. Daniels, Farkas, J. B. Foresman, J. V. Ortiz, J. Cioslowski, D. J. Fox, *Gaussian 09, Revision D.01*, **2009**.

[5] G. J. Martyna, M. L. Klein, M. Tuckerman, *J. Chem. Phys.* **1992**, *97*, 2635–2643.

[6] N. Troullier, J. L. Martins, *Phys. Rev. B* **1991**, *43*, 1993–2006.

[7] W. Humphrey, A. Dalke, K. Schulten, *J. Molec. Graphics* **1996**, *14*, 33.

[8] N. S. Zefirov, V. A. Palyulin, E. E. Dashevskaya, *Journal of Physical Organic Chemistry* **1990**, *3*, 147–158.

[9] D. Cremer, J. A. Pople, *Journal of the American Chemical Society* **1975**, *97*, 1354–1358.

[10] C. Altona, M. Sundaralingam, *Journal of the American Chemical Society* **1972**, *94*, 8205–8212.

[11] S. J. Han, Y. K. Kang, *Journal of Molecular Structure: THEOCHEM* **1996**, *369*, 157 – 165.

[12] O. V. Shishkin, P. Dopieralski, I. V. Omelchenko, L. Gorb, Z. Latajka, J. Leszczynski, *The Journal of Physical Chemistry Letters* **2011**, *2*, 2881–2884.

[13] O. V. Shishkin, P. Dopieralski, I. V. Omelchenko, L. Gorb, Z. Latajka, J. Leszczynski, *Journal of Molecular Modeling* **2013**, *19*, 4073–4077.

[14] L. Walewski, P. Dopieralski, O. V. Shishkin, Z. Latajka, *International Journal of Quantum Chemistry* **2014**, *114*, 534–542.

[15] I. V. Omelchenko, O. V. Shishkin, L. Gorb, J. Leszczynski, *Structural Chemistry* **2016**, *27*, 101–109.

[16] P. B. Liescheski, D. W. Rankin, *Journal of Molecular Structure* **1989**, *196*, 1–19.
